# Benchmarking SILAC Proteomics Workflows and Data Analysis Platforms

**DOI:** 10.1016/j.mcpro.2025.100980

**Published:** 2025-04-30

**Authors:** Ashley M. Frankenfield, Kevin L. Yang, Wan Nur Atiqah binti Mazli, Jamison Shih, Fengchao Yu, Edwin Lo, Alexey I. Nesvizhskii, Ling Hao

**Affiliations:** 1 Department of Chemistry, George Washington University, Washington, District of Columbia, USA; 2 Department of Computational Medicine and Bioinformatics, University of Michigan, Ann Arbor, Michigan, USA; 3 Department of Chemistry & Biochemistry, University of Maryland, College Park, Maryland, USA; 4 Department of Pathology, University of Michigan, Ann Arbor, Michigan, USA; 5 Data Science Institute, University of Chicago, Chicago, Illinois, USA

**Keywords:** DDA, DIA, protein turnover, proteomics data analysis, SILAC

## Abstract

Stable isotope labeling by amino acids in cell culture (SILAC) is a powerful metabolic labeling technique with broad applications and various study designs. SILAC proteomics relies on the accurate identification and quantification of all isotopic versions of proteins and peptides during both data acquisition and analysis. However, a comprehensive comparison and evaluation of SILAC data analysis platforms is currently lacking. To address this critical gap and offer practical guidelines for SILAC proteomics data analysis, we designed a comprehensive benchmarking pipeline to evaluate various *in vitro* SILAC workflows and commonly used data analysis software. Ten different SILAC data analysis workflows using five software packages (MaxQuant, Proteome Discoverer, FragPipe, DIA-NN, and Spectronaut) were evaluated for static and dynamic SILAC labeling with both DDA and DIA methods. For benchmarking, we used both in-house generated and repository SILAC proteomics datasets from HeLa and neuron culture samples. We assessed 12 performance metrics for SILAC proteomics including identification, quantification, accuracy, precision, reproducibility, filtering criteria, missing values, false discovery rate, protein half-life measurement, data completeness, unique software features, and speed of data analysis. Each method/software has its strengths and weaknesses when evaluated for these performance metrics. Most software reaches a dynamic range limit of 100-fold for accurate quantification of light/heavy ratios. We do not recommend using Proteome Discoverer for SILAC DDA analysis despite its wide use in label-free proteomics. To achieve greater confidence in SILAC quantification, researchers could use more than one software packages to analyze the same dataset for cross-validation. In summary, this study offers the first systematic evaluation of various SILAC data analysis platforms, providing practical guidelines to support decision-making in SILAC proteomics study design and data analysis.

## Highlights

• Five software tools with DDA and DIA workflows were benchmarked for SILAC proteomics.
• Static and dynamic SILAC were evaluated for protein quantification and turnover.
• SILAC proteomics is unable to accurately quantify differences greater than 100-fold.
• Removing low-abundant peptides and outlier ratios improves SILAC quantification.
• Selecting appropriate labeling time points is crucial for dynamic SILAC experiments.

## Graphical Abstract

**Figure:**
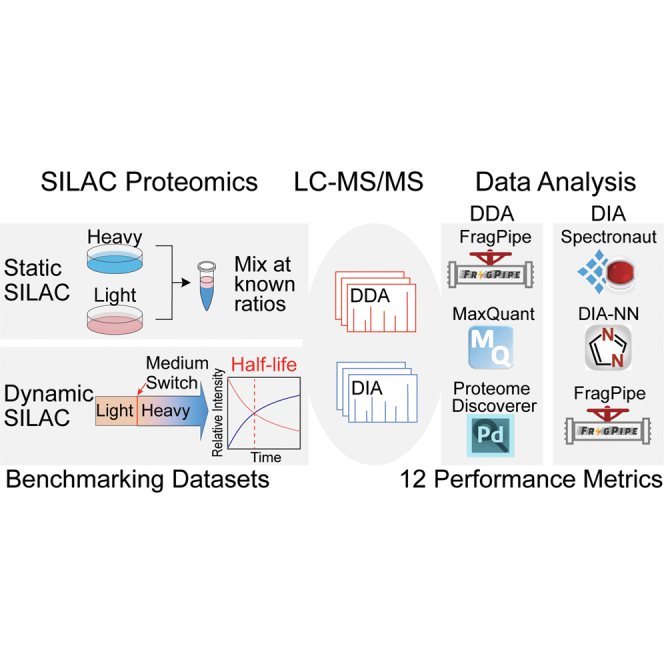

## In Brief

This benchmarking study evaluated various SILAC proteomics workflows using five proteomics software packages: FragPipe, MaxQuant, DIA-NN, Spectronaut, and Proteome Discoverer. Twelve performance metrics were assessed for both static and dynamic SILAC labeling, as well as DDA and various DIA data analysis modes.

Stable isotope labeling by amino acids in cell culture (SILAC) is a powerful technique for the quantitative analysis of proteins (1, 2). Light or heavy amino acids can be incorporated into proteins during translation to generate known mass shifts in peptides and proteins that can be detected and quantified by mass spectrometry (MS) platforms. The SILAC technique has been widely used in quantitative proteomics, affinity purification, studying post-translational modification, and measuring protein kinetics (3, 4, 5, 6, 7). Besides traditional static SILAC, dynamic SILAC (dSILAC) labeling can be used to measure protein turnover by switching the light amino acids to heavy (or heavy to light) from the nutrient source at a predefined time point *in vitro* or *in vivo* (8, 9, 10, 11). Newly synthesized proteins incorporate the heavy amino acids while the pre-existing light proteins will be degraded over time, and protein turnover rates can be calculated based on the heavy to light ratios at specific time points after the medium switch. Compared to label-free proteomics, SILAC proteomics provides multiplexing capabilities to reduce experimental variations, improve quantification accuracy, and remove false-positive interactions (12, 13).

SILAC samples and datasets contain multiple isotopic versions of the same proteome with increased MS spectral complexity compared to label-free proteomics. A major challenge for SILAC proteomics is to confidently identify and quantify all isotopic versions of the peptides and proteins. Since the development of the MaxQuant (14) software for SILAC proteomics, various proteomics software platforms have expanded the capability to analyze SILAC data (15, 16, 17, 18, 19). With the recent technological advances in data-independent acquisition (DIA) methods and computational tools, SILAC proteomics can now be conducted with both data-dependent acquisition (DDA) and DIA pipelines (20, 21, 22, 23, 24). Dynamic SILAC data adds another layer of complexity for modeling SILAC data with different time points and changing trend. However, there are no clear guidelines on SILAC data processing, filtering, software parameter settings, and evaluation criteria to ensure SILAC data quality in DDA and DIA pipelines and in static and dynamic SILAC workflows.

Although various benchmarking studies have been conducted for label-free proteomics, they cannot be directly used to evaluate SILAC data analysis (25, 26, 27, 28). A comprehensive comparison and evaluation of SILAC data analysis platforms is still lacking. Optimizing separation and MS methods can improve the proteome coverage and reduce missing values in SILAC data, but the proper selection and use of proteomics software are perhaps more important to successfully identify and quantify the multiple isotopic versions of co-eluting peptide peaks. Given the wide application of SILAC labeling in biological studies, SILAC data analysis should be made accessible to nonexpert users. Clear data analysis guidelines are thus important to ensure high data quality and true biological discoveries.

In this study, we conducted a comprehensive benchmarking of various SILAC data analysis workflows (static SILAC, dynamic SILAC, DDA, library-based DIA, hybrid-DIA, direct DIA) using different proteomics software (MaxQuant (14), Proteome Discoverer (PD), FragPipe (19), Spectronaut (17), and DIA-NN (16)). We evaluated 12 performance metrics for SILAC proteomics including identification, quantification, accuracy, precision, reproducibility, filtering criteria, missing values, false discovery rate, protein half-life measurement, data completeness, unique software features, and speed of data analysis. We offer practical suggestions and guidelines for users to design the SILAC workflow and choose the most appropriate data analysis platforms based on the characteristics of the dataset and custom needs.

## Experimental Procedures

### Experimental Design and Statistical Rationale

This study aims to compare different SILAC proteomics data analysis workflows for static and dynamic SILAC proteomics with both DDA and DIA methods. Both in-house generated and repository SILAC proteomics datasets were used for benchmarking with more than 400 raw data files from HeLa and inducible pluripotent stem cell (iPSC)-derived neuron samples. The datasets were used to evaluate protein/peptide identification, quantification, reproducibility, false discovery, half-life measurements, data filtering criteria, and unique software features for SILAC and dSILAC proteomics workflows. Various SILAC proteomics software platforms (Maxquant, Proteome Discoverer, FragPipe, DIA-NN, Spectronaut) and different data processing modes were compared for the analysis of DDA and DIA (library-based, hybrid, direct DIA) SILAC proteomics data. Triplicate technical replicates were used for DDA/DIA static SILAC data. Four time points with three biological replicates were used in the DDA dSILAC data. Highly fractionated samples were used in the DIA dSILAC data to provide a comprehensive neuron proteome spectral library. For the library-based DIA analysis, three spectral libraries were generated using the DDA raw files from the HeLa and iPSC-derived neuron samples. For the hybrid-DIA analysis, additional hybrid libraries were created using the DDA spectral library, and the DIA raw files were used for quantification. Detailed information of the benchmarking datasets and spectral libraries are summarized in Supplemental Table S1. Statistical analysis was performed in R using a two-tailed student *t* test, root-mean squared error, and mean absolute deviation.

### SILAC Cell Culture and Proteomics Sample Preparation

Cellular samples and proteomics data from multiple human cell lines were used in this study, including HeLa and human iPSC-derived cortical neurons. The routine cell culture steps have been described elsewhere (22, 29, 30). Heavy cell culture medium for each cell line was prepared by supplementing heavy lysine (^13^C_6_^15^N_2_) and/or arginine (^13^C_6_^15^N_4_) in lysine- and arginine-deficient medium. For traditional SILAC labeling in dividing cells (HeLa), complete incorporation of heavy amino acids was confirmed after multiple days of culture with at least five doubling times before harvesting. For dSILAC labeling in neurons, human iPSCs were differentiated into glutamatergic cortical neurons in normal light medium and switched to heavy lysine-containing medium after neuron maturation as described previously (10). Neurons were harvested at multiple time points (0, 1, 2, 4, 6-days) after the heavy medium switch.

The cells were lysed in ice-cold lysis buffer (8 M Urea, 50 mM Tris, 150 mM NaCl) and sonicated using a QSonica Q700 sonicator with 1 min on, 30 s off cycles for 15 min in an ice-cold water bath. The cell lysates were clarified by centrifugation, followed by total protein concentration measurement and bottom-up proteomics steps as described previously (10, 29). Reduced and alkylated SILAC samples labeled with both heavy lysine and arginine were digested with Trypsin/LysC mix (Promega) with an enzyme: protein ratio of 1: 20 (μg) for 18 h at 37 °C. SILAC samples labeled with only heavy lysine were digested with LysC enzyme (Promega) under the same digestion conditions. The samples were quenched by 10% TFA to pH < 3 and desalted by Oasis HLB 96-well plate (Waters). All the samples were dried under a SpeedVac and stored at −30 °C until LC-MS analysis.

### DDA and DIA LC-MS/MS Analysis

All peptide samples were reconstituted in 2% acetonitrile with 0.1% formic acid in water and clarified by centrifugation. LC-MS/MS acquisition was conducted using a Dionex Ultimate 3000 RSLCNano system coupled to a Thermo Fisher Q-Exactive HF-X mass spectrometer. The peptides were loaded onto an Acclaim PepMAP C18 trap column (3 μm, 100 Å, 75 μm × 2 cm) and separated by an Easy-spray PepMAP RSLC C18 column (2 μm, 100 Å, 75 μm × 75 cm) maintained at 55 °C. The DDA data were collected by performing a precursor scan (*m/z* 400–1000) at 120K resolution. An automatic gain control (AGC) target of 1 × 10^6^ and a maximum injection time (MaxIT) of 60 ms were used. The top 40 precursors were selected with a 1.4 Da isolation window and fragmented with a normalized collision energy of 30%. MS/MS spectra were collected at a 7.5 K resolution with an AGC of 2 × 10^5^ and a MaxIT of 40 ms. The DIA data were collected by performing a precursor scan (*m/z* 400–1000) at 60K resolution. MS1 AGC was set to 1 × 10^6^, and MaxIT was set to 60 ms. MS/MS spectra were collected using an overlapped, 8 Da isolation window scheme at a 15K resolution with a normalized collision energy of 30%, an AGC of 2 × 10^5^, and a MaxIT of 40 ms.

### Proteomics Data Analysis Using Various Software Platforms

The DDA and DIA SILAC proteomics data were analyzed by a variety of software platforms using default settings and the following common parameters unless otherwise noted. A reviewed Swiss-Prot *Homo sapiens* database containing 20,402 protein entries (downloaded December 5, 2022) and our custom contaminant FASTA libraries (31) (available to download at https://github.com/HaoGroup-ProtContLib) were used with a 1% false discovery rate (FDR) for protein and peptide identification. A maximum of two missed cleavages was allowed. A fixed modification of cysteine carbamidomethyl and variable modifications of methionine oxidation and protein N-terminus acetylation were included. Precursor and fragment tolerances were both set to 20 ppm. The peptide length was set to 7 to 52 amino acids. For DDA data, b-type and y-type ions with up to two charges were used for quantification. For DIA data, only y-type product ions were used for quantification. Lys8 or Lys8/Arg10 was selected as the heavy SILAC channel. The output peptide-level data from each software was processed in Python or R for further comparison and evaluation. Peptides with an intensity below 1000 and all contaminant peptides were removed from the dataset.

#### MaxQuant

DDA SILAC data analyses were conducted by MaxQuant (v. 1.6.14) with standard multiplicity (*i.e*., two labels) and default settings (24). The contaminant library within MaxQuant was disabled and replaced with our custom contaminant library during data processing (31). Requantification was enabled to allow match between channels (MBC). Software calculated ratios from the peptide.txt file were used for further analysis.

#### Proteome Discoverer

DDA SILAC data analyses were conducted by PD (Thermo Fisher Scientific, v. 2.4.1.15) using a 2-plex quantification method with the light channel selected as the control. Under the Sequest HT node of the processing workflow, heavy isotopic channels were selected as potential variable modifications. Only high confident peptides (FDR <1%) were included in the report file. Minora, a match-between-run (MBR) function in PD, cannot be disabled in this software. For a fair comparison, we removed the peptides when both the heavy and light isotopic peak were found using this matching function.

#### FragPipe

FragPipe (v.20.0) was used for both DDA and DIA (library-based, hybrid, and direct DIA mode) SILAC data analyses. All the files were converted to an open-format (.*mzML*) using the msConvert feature of the ProteoWizard package (32) and recommended settings. For both the DDA and DIA analyses, the human and contaminant FASTA databases were combined into a single FASTA file, and Philosopher was used to generate target:decoy = 1:1 (33). MSFragger 3.8 was used for all database searches (19, 34, 35). For the DDA data analysis, the raw files were processed using an adapted “SILAC3” workflow with the medium channel removed. MSBooster 1.1.11 and Percolator 3.06.0 were enabled to perform PSM rescoring with spectral and retention time similarities (36, 37). ProteinProphet (38) was used for protein inference, and Philosopher (33) 5.0.0 was used for FDR filtering with the “-sequential” flag (i.e. applying PSM/peptide/ion level 1% FDR in addition to the 1% protein level FDR). IonQuant 1.9.8 was used with requantification enabled (39). The combined_modified_peptide_label_quant.tsv file was used as the report file. For DIA analysis, staggered DIA raw files were first demultiplexed with a mass error of 10 ppm to account for overlapping fragmentation windows. DIA raw files and all spectral libraries used the pre-made “DIA_SpecLib_Quant” workflow. Spectral libraries were created using EasyPQP (40). The plex DIA panel in the DIA-NN tab (version 1.8.2_beta_8) in FragPipe was enabled. With this option, FragPipe generated spectral libraries containing spectra of light peptide ions only. In doing so, the spectra of peptides identified in the heavy form only were converted to the light form (for peptide ions identified in both light and heavy form, the light form spectrum was used). This effectively enables the match between channels strategy for increasing the number of peptides identified in both light and heavy form (40). DIA-NN performed quantification with the FragPipe-generated library. When running DIA-NN, the library was not replaced by the predicted intensities. The report.tsv file (renamed as diann-output.tsv in FragPipe) was used as the report file. Precursors with Channel.Q.Value > 1% and Translated.Quantity <0 were removed from the dataset. Peptide abundance was determined by adding together the precursor abundance found under the “precursor.translated” column. Heavy to light ratios were calculated manually.

#### Spectronaut

Spectronaut (Biognosys, v.18.1) was used for DIA SILAC data analysis (library-based, hybrid, and direct modes). The raw files were directly imported into Spectronaut using the common parameters as described above. The spectral libraries were created directly in the Pulsar module (17). MBC was enabled by selecting “in-silico generation of missing channels” for a labeled workflow. Light intensity is denoted as the “reference” and heavy intensity is the “target.” The data was filtered using a precursor q-value <1% and a protein q-value (experiment) < 1%). A precursor-level report was used for further analysis. Precursor abundances were combined to determine peptide-level abundance. Heavy to light ratios were calculated in R.

#### DIA-NN

DIA-NN (v. 1.8.1) was used as a stand-alone tool (in addition to using it as part of FragPipe) for direct DIA SILAC data analysis. The DIA *.mzML* files from msConvert were loaded into DIA-NN. First, a predicted spectral library was generated. For the Lys8 samples, the predicted library was loaded and the following “additional options” were added: “--original-mods --peak-translation --fixed-mod SILAC,0.0,K,label --lib-fixed-mod SILAC --channels SILAC,L,K,0;SILAC,H,K,8.014199.” Samples labeled with Arg10/Lys8 were quantified using the following “additional options”: “--peak-translation --original-mods --fixed-mod SILAC,0.0,KR,label --lib-fixed-mod SILAC --channels SILAC,L,KR,0:0; SILAC,H,KR,8.014199:10.008269.” Isotopologs and no shared spectra were enabled. The report.tsv was used for further analysis. Precursors with Channel.Q.Value > 1% and Translated.Quantity <0 were removed from the data set. Peptide abundance was determined by adding together the precursor abundance from the “precursor.translated” column. Heavy to light ratios were manually calculated.

### Estimating FDR with an Entrapment Database

FDR was evaluated using an entrapment FASTA database that contained 48,040 proteins from several diverse species (20,402 *H. sapiens* proteins, 16,381 *Arabidopsis thaliana* proteins, 6727 *Saccharomyces cerevisiae* proteins, 4530 *Escherichia coli* proteins) (41). The false discovery proportion (FDP) was estimated using the equation (1): $FDP=\frac{N_{h}}{N_{0}}\times \frac{n_{0}}{n_{h}}$, where N_h_ and N_0_ are the number of *H. sapiens* and non-*H. sapiens* proteins in the entrapment database, respectively, and n_h_ and n_0_ are the number of *H. sapiens* and non-*H. sapiens* proteins quantified, respectively.

### Protein Half-Life Measurement

Global peptide half-lives were calculated using the heavy/light peptide ratios from the dSILAC proteomics data. Peptide abundances below 1000 and peptides attributed to contaminant proteins were removed from the dSILAC datasets. Only proteins and peptides with FDR <1% were used. The heavy-to-light peptide ratio from each sample was calculated. Ratios below 0.01 or above 100 were removed. Relative isotope abundance (RIA) was calculated using the equation (2): $RIA=\frac{R}{1+\left(R\right)}$, where R is the heavy-to-light ratio. The RIA for each time point was then fit with an exponential decay model, equation (3): $RIA=e^{-k_{loss}t}$, where *t* is the time point and *k*_*loss*_ is the rate of protein decay. Peptide half-life was determined as the time when 50% of peptides were degraded. Half-lives quantified with less than three time points or which had curve fitting R^2^ < 0.8 were removed from the dataset. The apparent protein half-life was calculated as the harmonic mean of all the unique peptides belonging to a specific protein.

### Statistical Analysis

All statistical analyses and data visualizations were conducted in R (v. 3.6) or Origin Lab, unless otherwise noted. A two-tailed Student’s *t* test was used to measure the difference between two groups. Root-mean squared error was used to measure the average distance between a predicted and calculated value. Mean absolute deviation was used to measure the average distance between the observed and mean value, where larger results signify greater variance. Spearman’s correlation coefficients were calculated in R.

## Results

### Establishing a Benchmarking Workflow for SILAC Proteomics

**Fig. 1:**
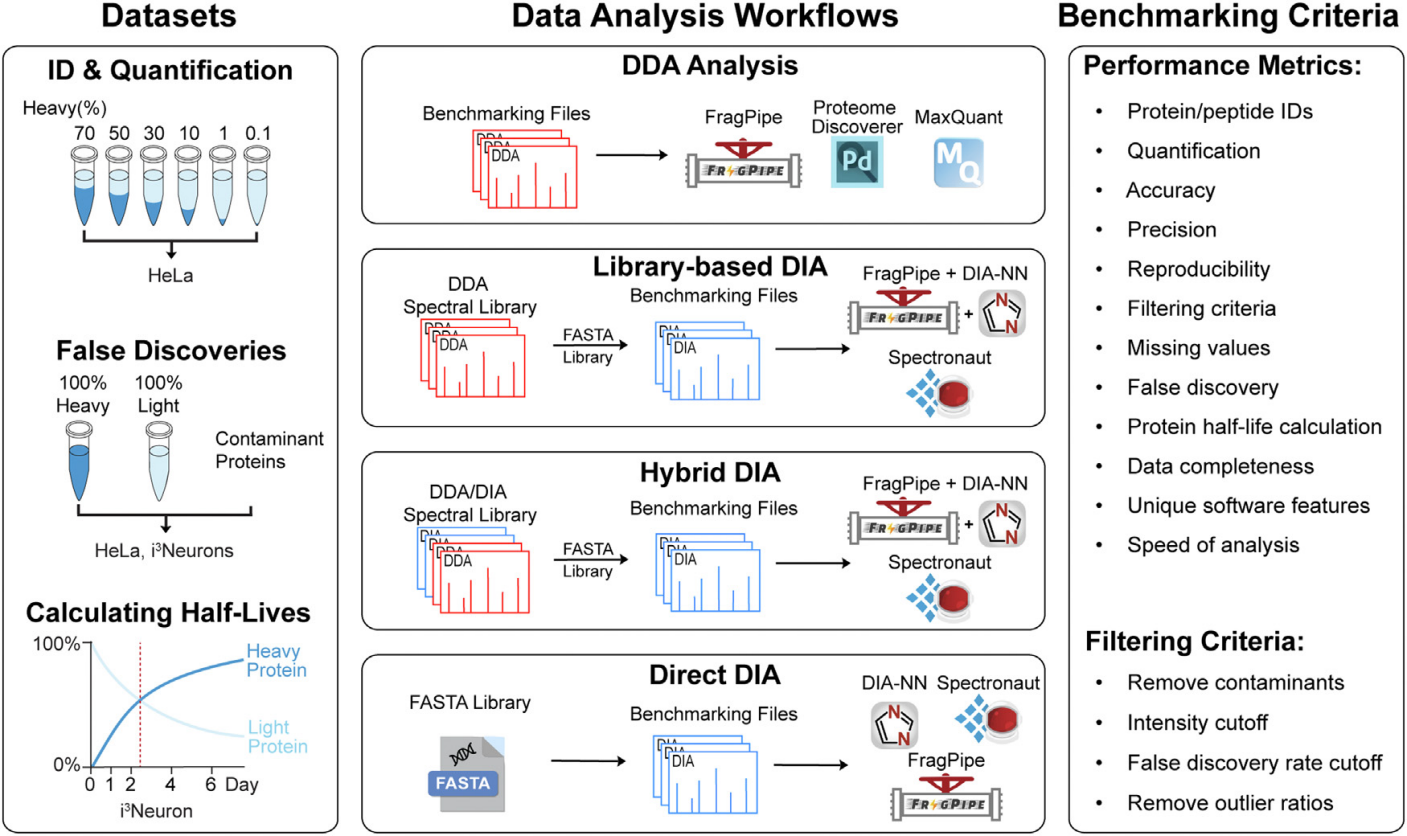
**Overall benchmarking workflow for SILAC proteomics.** The performance of static and dynamic SILAC proteomics workflows and various data acquisition and data analysis software platforms were evaluated using both in-house and repository SILAC proteomics datasets. The DDA proteomics data were analyzed with FragPipe, Proteome Discoverer (PD), and MaxQuant software. The DIA proteomics data were analyzed with FragPipe, DIA-NN, and Spectronaut software in three modes: library-based DIA (LB-DIA), hybrid DIA (H-DIA), and direct DIA (D-DIA). FragPipe uses DIA-NN to perform DIA quantification.

A comprehensive benchmarking workflow was designed to evaluate different SILAC systems and data analysis platforms (Fig. 1). Static and dSILAC proteomics workflows were respectively evaluated for abundance quantification and protein half-life measurements. The DDA proteomics data was analyzed with FragPipe, Proteome Discoverer, and MaxQuant. Library-based (LB)-DIA and hybrid (H)-DIA were conducted by first generating spectral libraries in FragPipe and Spectronaut and then performing further DIA data analyses. Direct (D)-DIA was conducted by directly analyzing DIA files against a protein sequence database in FragPipe, Spectronaut, and DIA-NN. The datasets used for benchmarking and the details of the spectral libraries generated here are provided in Supplemental Table S1.

### Evaluating Proteomics Identification and Quantification of SILAC Data Analysis Platforms

**Fig. 2:**
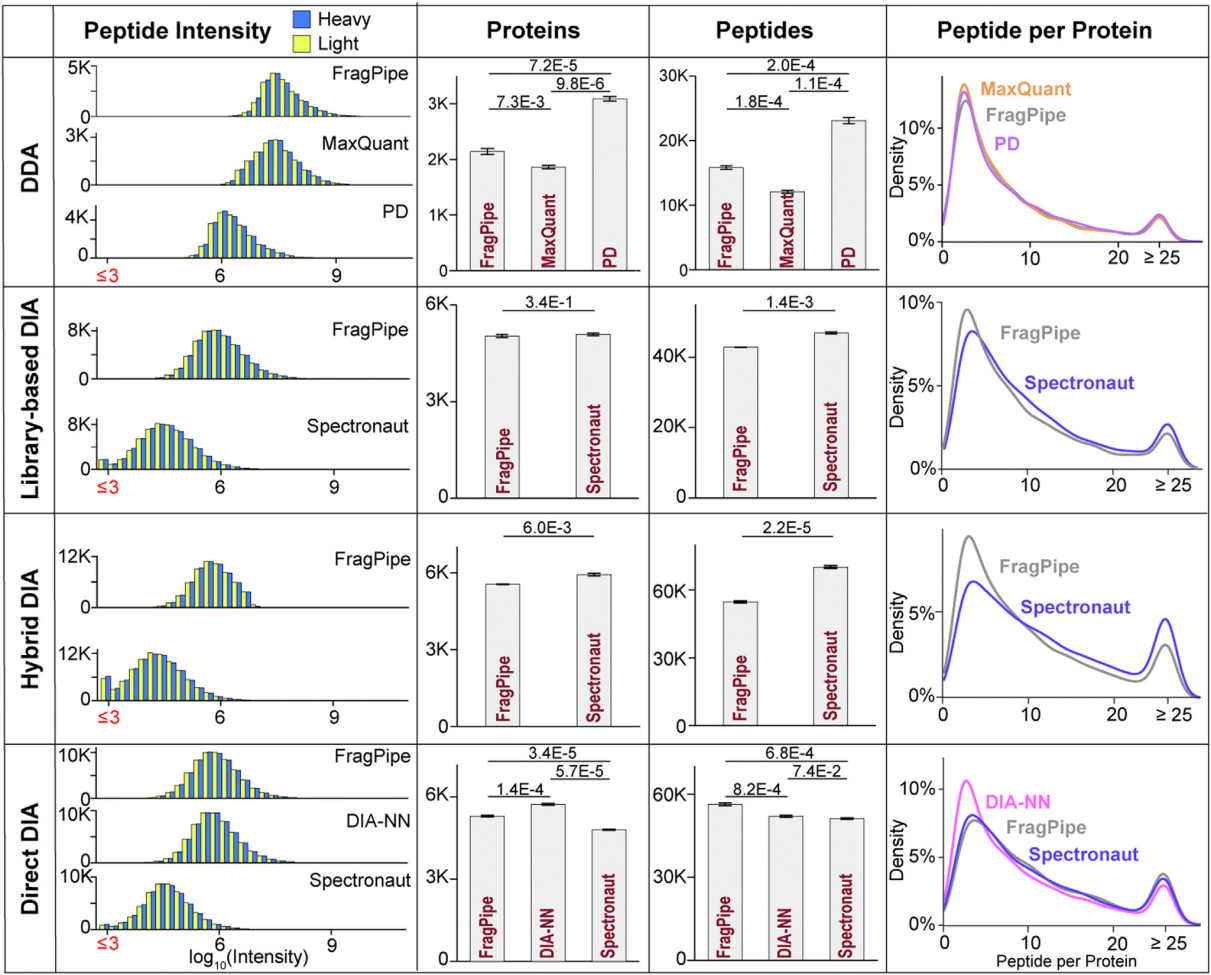
**Comparing protein and peptide identifications across different SILAC workflows and data analysis platforms.** Histograms showing the intensity distributions of quantified heavy and light peptides. Heavy and light peptides with intensities below 1000 are highlighted in Supplemental Fig. S1 and removed from subsequent analysis. Bar graphs show the number of proteins and peptides quantified across three technical replicates with a heavy-to-light isotopic ratio. *p*-values from a student’s *t* test are labeled on the graph. Match between channels (MBC) was enabled for FragPipe (DDA, LB-DIA, H-DIA, D-DIA), MaxQuant (DDA), and Spectronaut (LB-DIA, H-DIA, and D-DIA). Match between runs (MBR) was enabled by default in PD and DIA-NN (D-DIA). Density plots show the distribution of the number of unique peptides used to quantify each protein.

We first comparatively evaluated the proteome identification for the commonly used SILAC data analysis methods and software, including DDA (FragPipe, MaxQuant, Proteome Discoverer), LB-DIA (FragPipe, Spectronaut), hybrid-DIA (H-DIA) (FragPipe, Spectronaut), and direct-DIA (D-DIA) (FragPipe, DIA-NN, Spectronaut) (Fig. 2). SILAC DDA and DIA proteomics datasets from HeLa cells with a known 1:1 heavy/light ratio and three technical replicates were used for this comparison (22). Each software was run based on the developer’s recommended settings to mimic a common user’s experience. DDA uses MS^1^ precursors for quantification and therefore generates higher peptide intensities than the DIA method which uses MS^2^ fragment ions for quantification (Fig. 2). We found that peptides with intensities below 1000 in DIA results have MS^2^ signals lower or close to noise level intensities and distorted ratios, particularly from the Spectronaut results using an orbitrap MS (Supplemental Fig. S1, *A* and *B*). To avoid ratio distortion, we recommend removing peptides with intensities below 1000 from all DIA results. Most software’s default settings remove precursors with protein and peptide FDR higher than 1%. When analyzing DIA data using DIA-NN and FragPipe (which uses DIA-NN for extracting quantification), the software outputs 1% FDR filtered “matrix files”, as well as the report.tsv file filtered with 1% run-wise precursor FDR. When using the report.tsv file, users need to apply additional filters to remove precursors with a channel q-value >1% (Supplemental Fig. S1*C*). After removing peptides with intensity <1000 and precursors with a channel q-value >1%, the average number of proteins quantified with a heavy-to-light ratio follow the ranking, H-DIA > D-DIA > LB-DIA > DDA (Fig. 2, Supplemental Fig. S2). Spectronaut provided the most quantified peptides in L-DIA and H-DIA. DIA-NN provided the most quantified proteins in D-DIA. FragPipe provided the most quantified peptides in D-DIA. Although H-DIA provided the most peptide quantifications, it also generated more peptides with low intensities and more scattered ratios (Supplemental Fig. S1*A*)

**Fig. 3:**
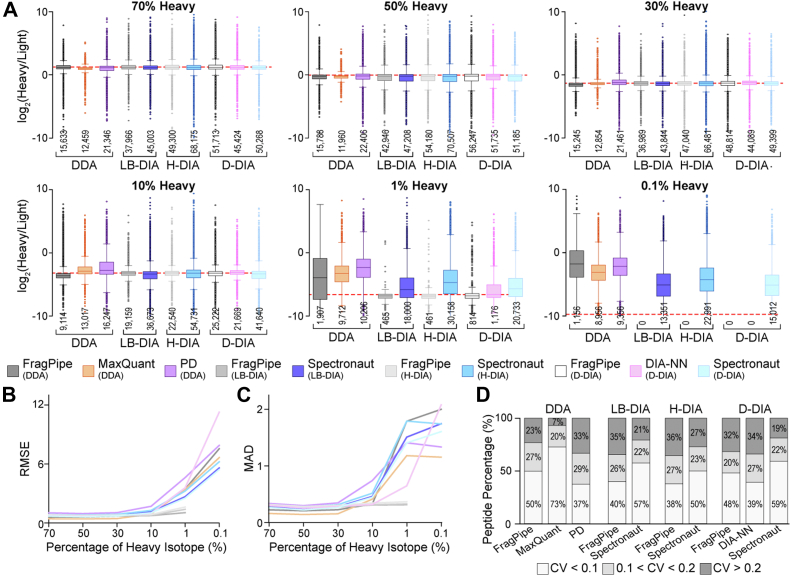
**Evaluating quantification accuracy and precision using a series of SILAC samples with known percentages of heavy peptides.***A*, box plots of quantified heavy/light ratios from SILAC samples with known percentages of heavy peptides from DDA, library-based DIA (LB-DIA), hybrid DIA (H-DIA), and direct DIA (D-DIA) SILAC data analysis platforms. The *dashed* line denotes the theoretical heavy/light ratios. The number below each boxplot indicates the number of peptides. *B*, RMSE showing quantification error in the different data analysis platforms. *C*, mean absolute deviation (MAD) showing quantification precision in the different data analysis platforms. *D*, stacked bar graphs showing the coefficient of variation (CV) across three technical replicates for SILAC samples with a 1:1 heavy/light ratio. CVs for other SILAC samples with various known percentages of heavy peptides are shown in Supplemental Fig. S3.

Next, we evaluated the quantification accuracy, precision, and reproducibility of different SILAC data analysis platforms with a series of SILAC samples with known heavy/light ratios (Fig. 3*A*). The peptide intensity and FDR filtering criteria mentioned above were applied before comparing the quantification performance. Most software platforms were able to accurately measure heavy/light SILAC ratios within a 10-fold difference (70%, 50%, 30%, and 10% heavy). DDA software exhibited less outlier ratios, indicating better confidence from MS1-level quantification. Particularly, DDA analysis with MaxQuant software showed the tightest clusters of peptide ratios (Fig. 3*A*). Among DDA results, PD provided the most protein/peptide quantifications but the least accurate and precise ratios. DIA outperformed DDA when the sample contained 1% heavy peptides. No software can accurately measure 0.1% heavy samples, indicating that a limit of quantification had been reached (Fig. 3*A*). Quantification precision and reproducibility among replicates also significantly dropped in the 1% and 0.1% heavy samples (Fig. 3, *B* and *C*, Supplemental Fig. S3). Therefore, SILAC proteomics can confidently quantify within 10-fold differences without ratio compression and the dynamic range of SILAC quantification should be controlled within 100-fold differences.

### Reducing Missing Values in SILAC Proteomics

**Fig. 4:**
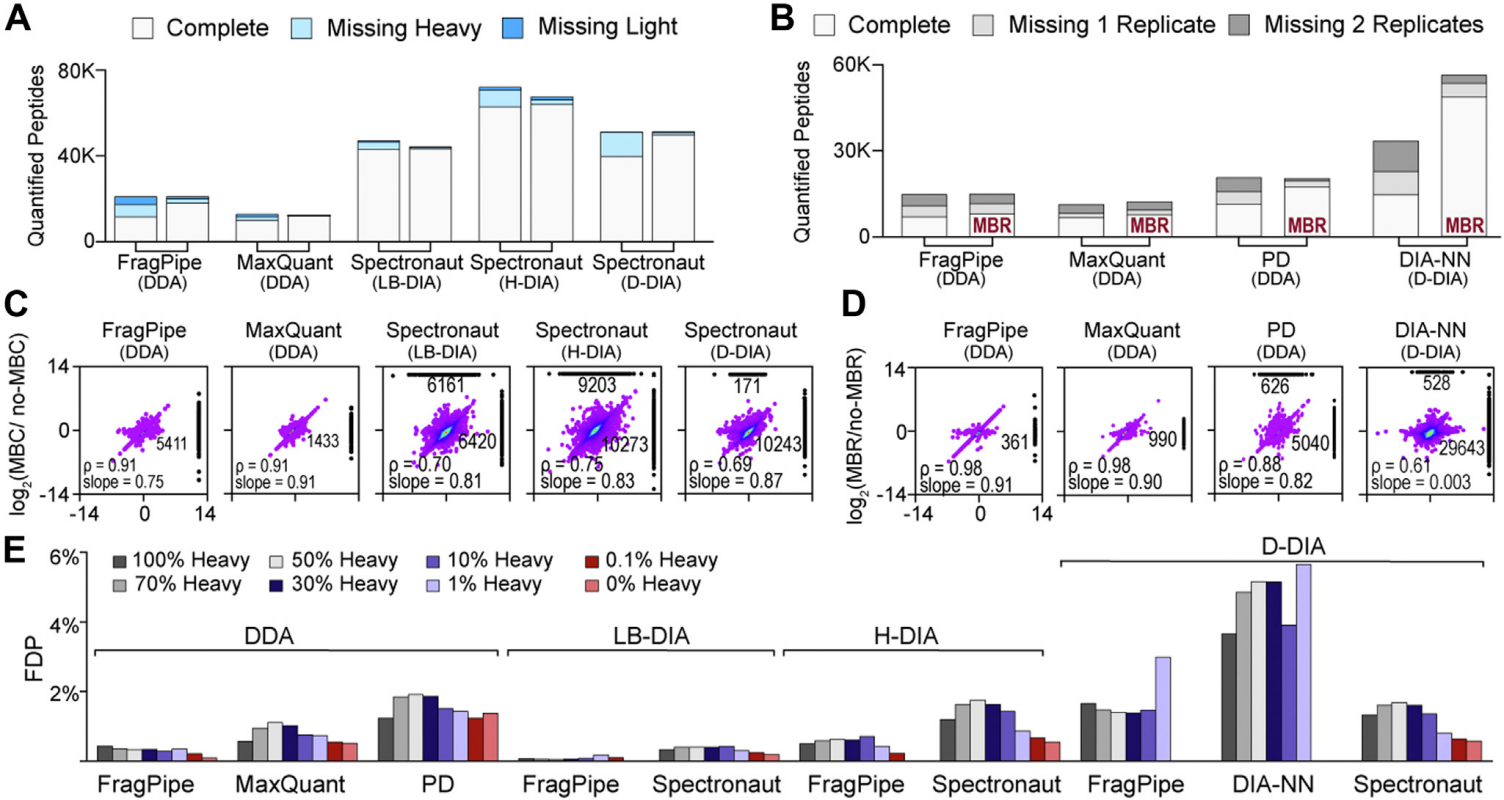
**Evaluating data analysis parameters to reduce missing values and false discoveries for SILAC proteomics.***A*, percentages of missing heavy/light channels with and without enabling the match between channels (MBC) function in different software. *B*, percentages of peptide missing values with and without enabling the match between run (MBR) function in different software. *C*, scatterplots showing Spearman’s correlation of peptide ratios with and without MBC. Correlation coefficients and slope are labeled on the graph. Dots on the *right* and *top* of the graph denote peptides that are only present with and without MBC, respectively. *D*, scatterplots showing the spearman’s correlation of peptide ratios with and without MBR. Quantification variations with and without MBC and MBR functions are shown in Supplemental Fig. S4. *E*, false discovery proportion (FDP) calculated using an entrapment method.

For SILAC samples, proteins and peptides can only be quantified when all isotopic versions are detected to obtain heavy to light ratios. Therefore, it is crucial to reduce missing values in SILAC proteomics, both for missing isotopic channels in the same peptide and missing ratios across multiple samples. To reduce missing isotope channels in DDA data analysis, users can check the “requantify” function in MaxQuant and FragPipe software. We refer to runs within a requantification step as MBC, in analogy with the MBR terminology. For DDA analysis, MaxQuant showed the least missing heavy/light channels, whereas PD results had 44% of peptides missing heavy or light peptides. For DIA analysis, Spectronaut provides the “in-silico generate missing channels” function. In FragPipe, the library that is passed to DIA-NN for quantification is generated containing the light form of the peptide ions only, regardless of whether the peptide was identified in light or heavy form. These strategies for DIA data, as with the requantification option for DDA data, were implemented to improve the quantification of peptides in both light and heavy form (thus, we refer to this as MBC). For DIA analysis, FragPipe has the lowest percentage of missing isotope channels. Spectronaut benefited the most from MBC with over 97% peptides with complete isotopic channels (Fig. 4*A*). Enabling MBC increased the number of quantified peptides. But we found that quantification of coefficient of variations may be negatively influenced by MBC, particularly in DDA SILAC data (Fig. 4*C*, Supplemental Fig. S4*A*).

To reduce the missing values across multiple samples, users can check the MBR function in MaxQuant, FragPipe (DDA), DIA-NN, and the Minora function in PD. DDA software performs MBR by transferring peptide identification between raw data files based on their mass-to-charge ratio and retention time, while DIA software creates sample-specific libraries during the first mass search and use this for quantification matching. When MBR was enabled, DIA platforms showed significantly fewer missing values than DDA platforms, thus enabling large-scale proteomics analysis. This is consistent with previous reports by others (16, 42, 43, 44). Direct DIA using Spectronaut resulted in the lowest percentage of missing values across multiple samples. DIA-NN particularly benefited from enabling MBR with significantly increased protein and peptide identifications (Fig. 4*B*). However, the appearance of data completeness (i.e. a lower number of missing values) may also be a sign of poorly controlled run-specific FDR. Indeed, the high variability across replicates is apparent in the Spectronaut results. Enabling MBR in DIA-NN seemed to alter the peptide ratios and increase the variations across replicates as well (Fig. 4*D*, Supplemental Fig. S4*B*). Therefore, we recommend that users enable the developer-recommended functions for SILAC proteomic data analysis but be cautious of their impact on reproducibility between replicates.

### Assessing False Discoveries in SILAC Proteomics

Besides identifying peptide sequences using MS/MS fragments as in label-free proteomics, SILAC proteomics also requires confident identification of different isotopic versions of peptides to ensure accurate quantification of heavy/light ratios. A unique feature of SILAC experiments is that contaminant proteins that have been introduced throughout the experimental workflow can be used to estimate false discoveries since contaminant proteins such as keratins, trypsin, and bovine proteins from the cell culture medium should only have the light peptide or protein. We found that DDA analysis using MaxQuant and FragPipe and DIA analysis using DIA-NN had significantly less false identification of heavy contaminant peptides compared to other methods (Supplemental Fig. S5). Next, we evaluated false discoveries using an entrapment approach, where protein databases are spiked with nonhuman proteins that are not present in the sample, such as *A. thaliana*, *S. cerevisiae*, and *E. coli* (41, 45). After data filtering, we calculated the FDP for each data analysis platform (Fig. 4*E*). LB-DIA exhibited the best performance across the various platforms, followed by DDA, H-DIA, and D-DIA methods. Interestingly, despite sharing quantification modules, DIA-NN showed significantly higher FDP in comparison to the FragPipe DIA workflows. All platforms exhibit less false discoveries with 0% heavy samples compared to 100% heavy. This is likely because light peptides are typically used as the reference channel in these software packages. Therefore, when a reverse labeling strategy is used in SILAC or when most peptides are heavy versions, we would recommend using LB-DIA or DDA with the FragPipe platform to minimize false discoveries.

### Characterizing Dynamic SILAC Proteomics Workflow to Measure Protein Turnover

**Fig. 5:**
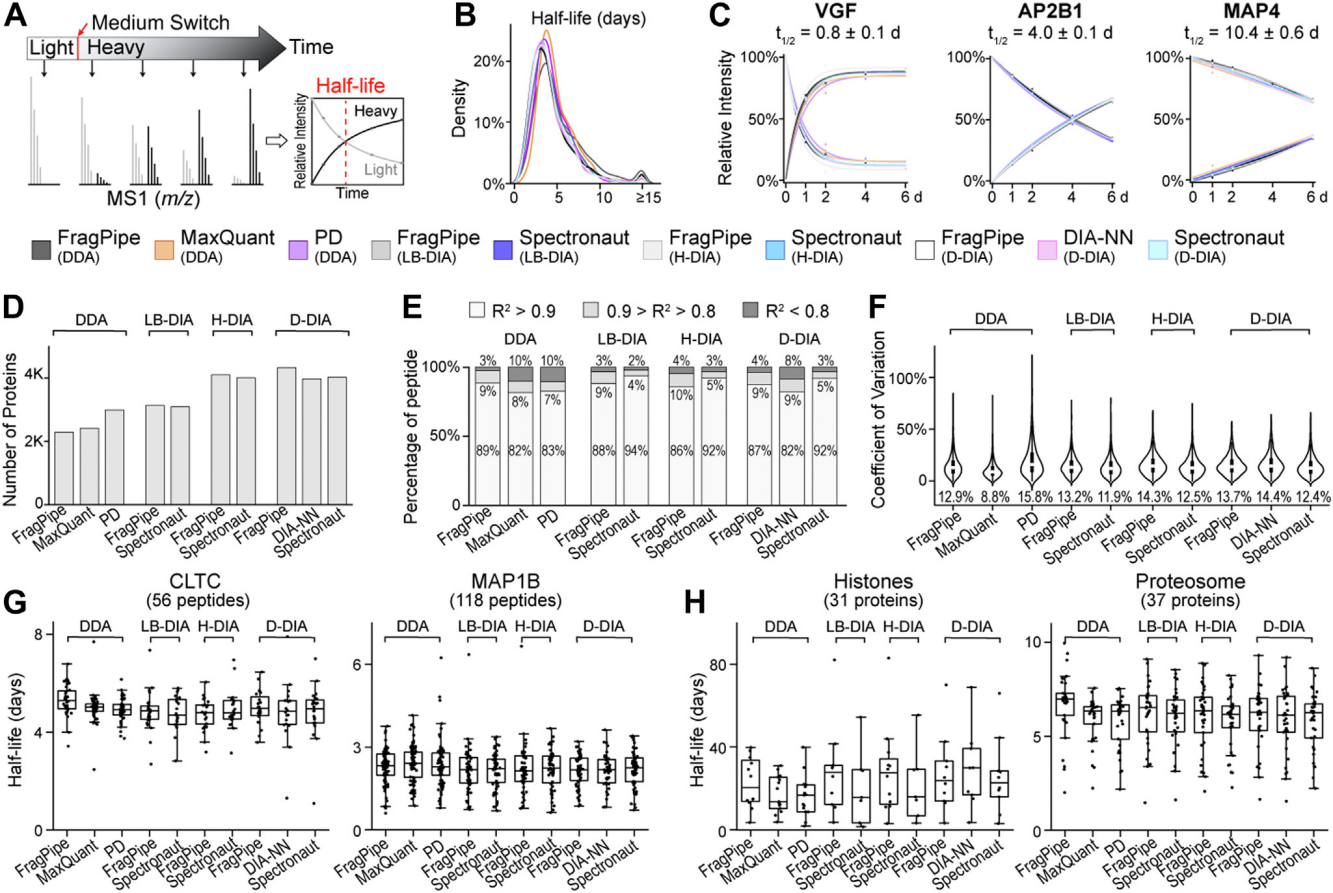
**Measuring global protein turnover using dynamic SILAC datasets in different data analysis platforms.***A*, schematics of dynamic SILAC (dSILAC) workflow to measure protein half-lives. *B*, density plot showing the distributions of global protein half-lives from different data analysis platforms. *C*, example protein degradation and synthesis curves showing fast, medium, and slow turnover proteins from human iPSC-derived neuron culture with 1, 2, 4, 6-days time points after medium switch. *D*, numbers of protein half-lives measured from different software. Numbers of measured peptide half-lives are provided in Supplemental Fig. S7. *E*, stacked bar chart showing the distribution of curve fitting correlation (R^2^) when fitting peptide degradation curves into an exponential decay model. *F*, violin plot distribution of coefficient of variation (CV) of half-lives from unique peptides belonging to the same protein. Median displayed below each plot. *G*, example box plot distributions of half-lives measured from unique peptides belonging to the same protein. *H*, example box plot distributions of protein half-lives measured for the same protein families.

Besides the static SILAC method that is mostly used for accurate protein quantification, dSILAC proteomics can measure global protein turnover in cells or animals (8). To evaluate the dSILAC workflows, we used our human iPSC-derived neurons with dSILAC labeling and multiple harvesting time points (0, 1, 2, 4, 6-days) after switching to a heavy medium for the comparison (Supplemental Table S1). Besides the filtering criteria established in traditional static SILAC data analysis, we found that dSILAC data contained outlier ratios that lead to outlier half-life values (Supplemental Fig. S6). Since no software can accurately quantify ratios below 1% heavy (Fig. 3), we removed peptides with heavy/light ratios below 0.01 and above 100 for all the software platforms. We found that different data analysis platforms calculated similar neuron protein turnover distributions with median half-lives around 4 days, consistent with our previous report (Fig. 5, *B* and *C*, Supplemental Fig. S7*A*) (10). DIA platforms measured more protein half-lives than DDA platforms, and FragPipe (DIA) measured the most protein half-lives after applying the stringent filtering criteria as described in the method section (Fig. 5*D*, Supplemental Fig. S7*B*). When fitting the multiple time point data into an exponential decay model, the FragPipe (DDA) platform outperformed all other software with over 95% peptides’ coefficient of determination (R^2^) being higher than 0.9, followed by Spectronaut DIA platforms (over 93% peptides’ R^2^ > 0.9) (Fig. 5*E*). Multiple time point curve fitting was conducted at the peptide level to calculate peptide half-lives, which were then averaged to generate the protein half-lives. All software platforms showed consistent half-lives for unique peptides from the same protein with the smallest variation for the MaxQuant platform (Fig. 5, *F* and *G*). When comparing biological replicates of dSILAC dataset, Spectronaut (DIA) and Proteome Discoverer (DDA) showed less variations among replicates (Supplemental Fig. S7*C*). Protein half-lives measured from different software correlated well with each other except for the PD software (Fig. 5*H*, Supplemental Fig. S8). Therefore, among all nine platforms, we would recommend MaxQuant (DDA), Spectronaut (LB-DIA, D-DIA), DIA-NN (D-DIA), and FragPipe (LB-DIA, D-DIA) for dSILAC data analysis.

### Practical Guidelines for SILAC Proteomics Data Analysis

**Fig. 6:**
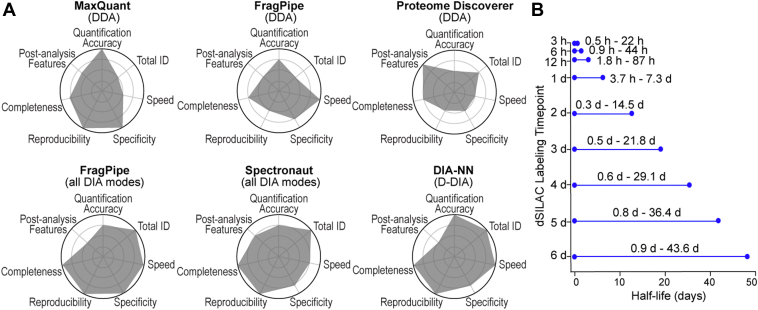
**Practical guideline for SILAC proteomics data analysis using different software packages.***A*, radar plots comparing the performance of different SILAC proteomics platforms to enable users to select software based on the quantification accuracy, total ID, speed, specificity, reproducibility, completeness of data, and postanalysis features. *B*, the range of accurate protein half-lives that can be measured when using different labeling time points for dynamic SILAC labeling.

After comprehensive evaluation of the SILAC proteomics workflows and data analysis platforms, we have summarized the performances of the different methods and software in Figure 6*A* for users to choose the most suitable platforms based on their specific requirements. We have also provided a summary of the software capabilities, features and recommended data analysis filtering criteria, and settings for static and dynamic SILAC proteomics data analysis in Supplemental Table S2. The quantified number of proteins/peptides follow the ranking H-DIA > D-DIA > LB-DIA > DDA. Although DDA provided the least proteome coverage, the SILAC DDA method provides better quantification precision and accuracy with less false discoveries compared with DIA methods, especially for low abundant peptides and proteins. For the DDA SILAC, we recommend using FragPipe or MaxQuant software due to its excellent quantification accuracy, reproducibility, and specificity. We do not recommend using PD for SILAC DDA analysis despite its wide use in label-free proteomics. For SILAC DIA platforms, FragPipe, Spectronaut, and DIA-NN provided comparable performance, each with its own advantages. FragPipe provided fast data processing and excellent reproducibility and specificity. Both FragPipe and Spectronaut provide versatile DIA capabilities (LB-DIA, H-DIA, D-DIA). The commercial Spectronaut software provides a more integrated, user-friendly interface for postdata analysis. DIA-NN provides excellent quantification accuracy for ratios requiring high dynamic range like 1% heavy samples. To reduce missing values in SILAC proteomics, MBC can be used to increase the number of quantifiable peptides with complete isotopic channels. Spectronaut particularly benefits from the MBC function. Enabling MBR reduced missing values across replicates particularly for large scale proteomics, particularly in the DIA-NN software. For SILAC proteomics, it is important to filter out the low abundance (<1000) and high FDR (>1%) peptides to ensure quantification accuracy. No software can accurately measure 0.1% heavy samples. Most software platforms reach the limit of dynamic range at a 100-fold difference. Outlier ratios (<0.01 or >100) should also be removed from dynamic SILAC dataset to accurately measure protein half-lives. We have also provided recommended labeling time points based on the range of the protein half-lives needed to be measured in custom experiments (Fig. 6*B*). For example, dividing cell types typically have a much faster turnover than postmitotic cells or animal tissues (11). Ubiquitin-related and secretory proteins have a much faster turnover, while histones and structural proteins have relatively slow turnover (11, 46). To ensure accurate measurement of protein half-lives, SILAC labeling experiments need to be designed with proper labeling time points based on the range of protein half-lives.

## Discussion

Despite the wide application of SILAC proteomics, a comprehensive comparison and evaluation of SILAC workflows and data analysis platforms is still lacking. Compared to label-free proteomics, SILAC proteomics generates more complex spectra with multiple isotopic channels that need to be identified and quantified with great confidence. In this study, we systematically benchmarked various SILAC workflows and software to provide practical guidelines for SILAC proteomics data analysis. Ten different data analysis workflows were evaluated for DDA and DIA methods including MaxQuant, PD, FragPipe, Spectronaut, and the DIA-NN software. We first compared the software using the developers’ recommended settings and then further evaluated unique software features and parameters such as MBC, MBR, filtering criteria, and FDR. Each method/software has its own pros and cons regarding identification, quantification, accuracy, precision, reproducibility, missing values, FDR, and speed of data analysis. We have provided practical suggestions and guidelines for users to choose the most appropriate software based on the characteristics of the dataset and custom needs. If researchers hope to achieve greater confidence in SILAC quantification results, two or more software packages can be used to analyze the same dataset for cross-validation.

We recognize that there are several limitations with this study. It represents a snapshot of several widely used software packages and their respective versions at the time of our data analysis. Besides the software platforms we evaluated in this study, other software can also be used for SILAC proteomics, such as Skyline (22), EncylopeDIA (18), and MetaMorpheus (9). New software and software versions will continue to be developed and can be evaluated using the same benchmarking design and parameters detailed in this study. Additionally, we focused on *in vitro* SILAC labeling in cell culture in this study. While the same principles apply for *in vivo* SILAC labeling in live animals, protein quantification and turnover measurements require a consideration for incomplete labeling and the recycling of intrinsic amino acids from protein degradation, as discussed elsewhere (47, 48, 49). Despite these limitations, this study represents the first comprehensive evaluation of the different SILAC workflows and data analysis platforms. The practical guidelines and benchmarking workflows in this study can benefit the broad field of SILAC proteomics.

## Data Availability

All LC-MS/MS data and spectral libraries generated in this study have been deposited to ProteomeXchange (50) Consortium (Identifier: PXD057850). The other published datasets used in this study were obtained from ProteomeXchange, as summarized in Supplementary Table S1. The contaminant protein libraries are summarized in Supplementary Table S3. All source code is available to download at: https://github.com/HaoGroup.

## Supporting Information

This article contains supporting information.

## Conflicts of Interests

A. I. N. and F. Y. receive royalties from the University of Michigan for the sale of MSFragger and IonQuant software licenses to commercial entities. All license transactions are managed by the University of Michigan Innovation Partnerships Office, and all proceeds are subject to the university technology transfer policy. The other authors declare no other competing interests.
