# Supplementary material for: Benchmarking SILAC Proteomics Workflows and Data Analysis Platforms
Source: Mol Cell Proteomics. 2025 Apr 30;24(6):100980. doi: 10.1016/j.mcpro.2025.100980 (PMC12159905; doi:10.1016/j.mcpro.2025.100980)
Supplement: Supporting Information [file mmc1.pdf]

Supplemental Information
Benchmarking SILAC Proteomics Workflows and Data Analysis Platforms

Ashley M. Frankenfield[1], Kevin Yang[2], Wan Nur Atiqah binti Mazli[3], Jamison Shih[1], Fengchao Yu[4], Edwin Lo[5], Alexey I. Nesvizhskii[2,4], Ling Hao[1,3*]

[1]Department of Chemistry, the George Washington University, Washington, DC, 20052, USA
[2]Department of Computational Medicine and Bioinformatics, University of Michigan, Ann Arbor, MI, USA
[3]Department of Chemistry & Biochemistry, the University of Maryland, College Park, MD, 20742, USA
[4]Department of Pathology, University of Michigan, Ann Arbor, MI, USA
[5]Data Science Institute, the University of Chicago, Chicago, IL, 60637, USA

*Corresponding author
Ling Hao, PhD.
Associate Professor
Department of Chemistry & Biochemistry
University of Maryland
E-mail: linghao1@umd.edu

Table of Contents

- **Supplemental Fig. S1**: Evaluating the filtering criteria for peptide intensity in DIA SILAC proteomics software.
- **Supplemental Fig. S2**: Venn diagrams of quantified proteins and peptides from different SILAC proteomics platforms.
- **Supplemental Fig. S3**: Comparing SILAC quantification precision across technical replicates in different software platforms.
- **Supplemental Fig. S4**: Comparing the quantification variation with and without enabling match between channels (MBC) and match between run (MBR) functions.
- **Supplemental Fig. S5**: Example false identification of contaminant proteins in SILAC proteomics dataset.
- **Supplemental Fig. S6**: Removing outlier heavy/light ratios from dynamic SILAC proteomics data in different software platforms.
- **Supplemental Fig. S7**: Comparing dynamic SILAC proteomics using different data analysis platforms.
- **Supplemental Fig. S8**: Spearman's correlation of global protein half-lives measured using various SILAC proteomics data analysis platforms.
- **Supplemental Table S1**: Summary of SILAC proteomics datasets used in this study and generated spectral libraries.
- **Supplemental Table S2**: Summary of software capabilities, features, and recommended settings for SILAC proteomics data analysis.
- **Supplemental Table S3**: Contaminant protein libraries used in this study including sample type specific libraries for general cell culture and neuron samples.
- **Supplemental Table S4**: Protein and peptide half-lives calculated from different software using multiple time point curve fitting.

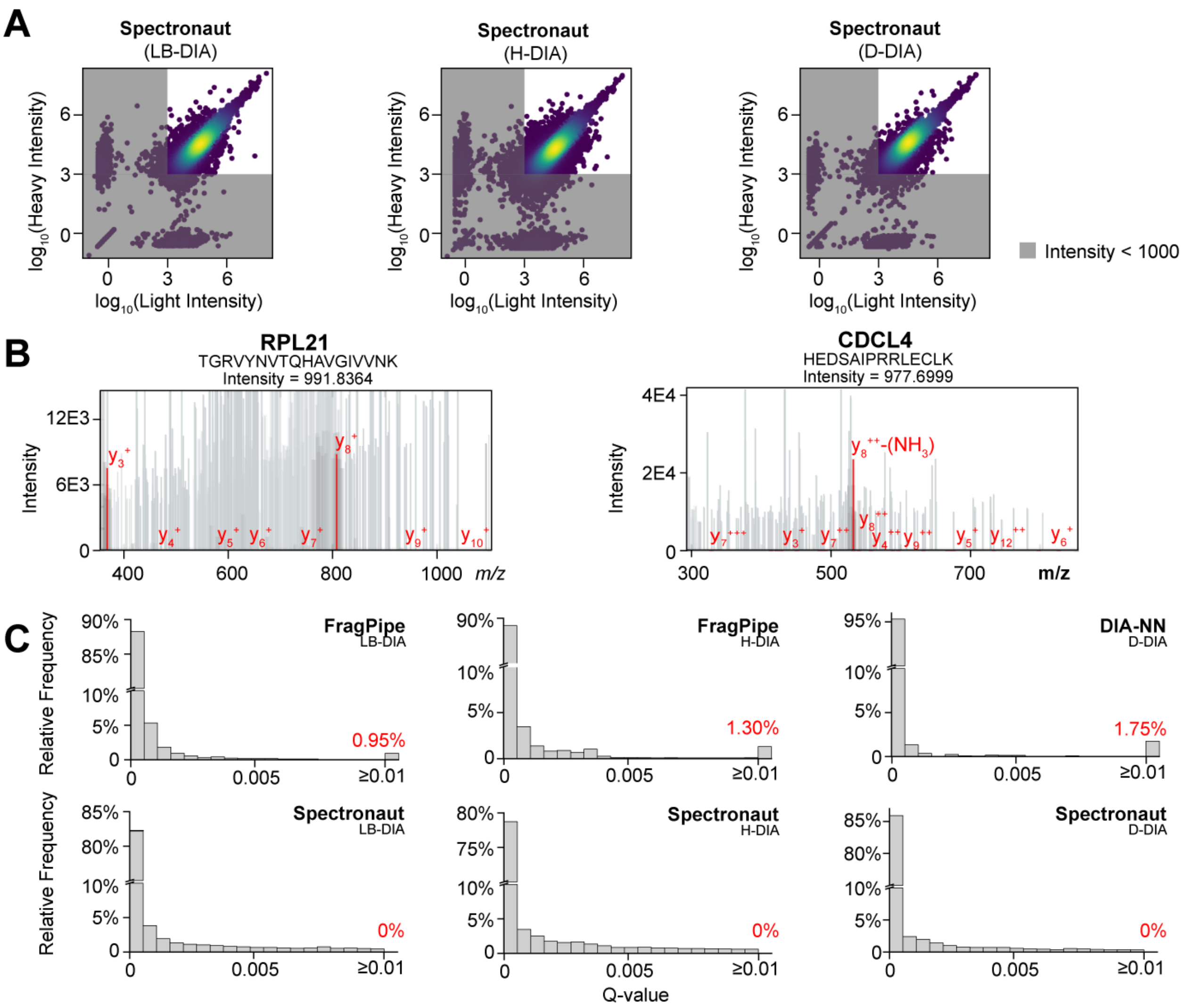

Supplemental Fig. S1: Evaluating the filtering criteria for peptide intensity in DIA SILAC proteomics software. A HeLa SILAC proteomics dataset with a known 1:1 heavy/light ratio and three technical replicates was used here. (A) Scatterplots showing the distribution and correlation of heavy and light peptide abundances. Peptides with intensities below 1000 were covered in gray shade and removed from subsequent analysis. (B) Example low abundant peptide MS/MS fragmentation spectra (intensity <1000) showing near or below noise level signals. (C) Histogram distributions showing the false discovery rate (FDR) of peptide identification from various software platforms. DIA peptide results from FragPipe and DIA-NN software are not filtered by FDR and need to be filtered by users after exporting the output files.

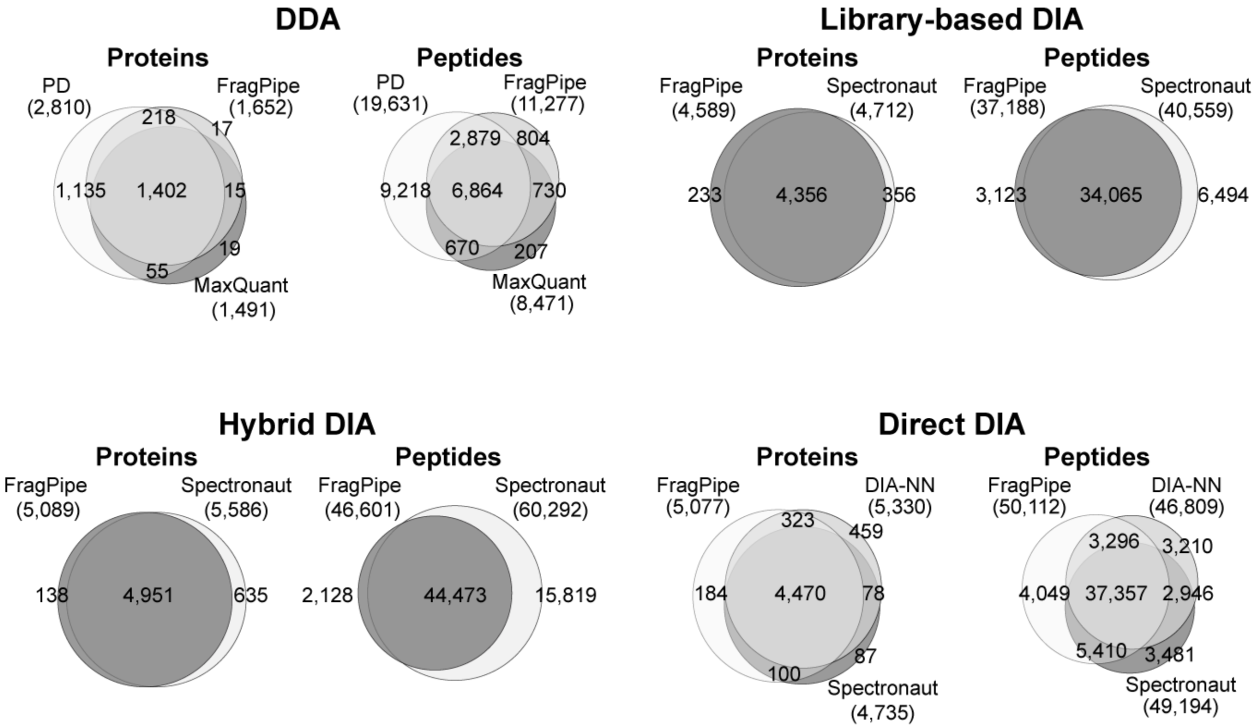

Supplemental Fig. S2: Venn diagrams of quantified proteins and peptides from different SILAC proteomics platforms. A SILAC proteomics dataset with a known 1:1 heavy/light ratio and three technical replicates was used here. Only proteins reproducibly quantified in three replicates were used here.

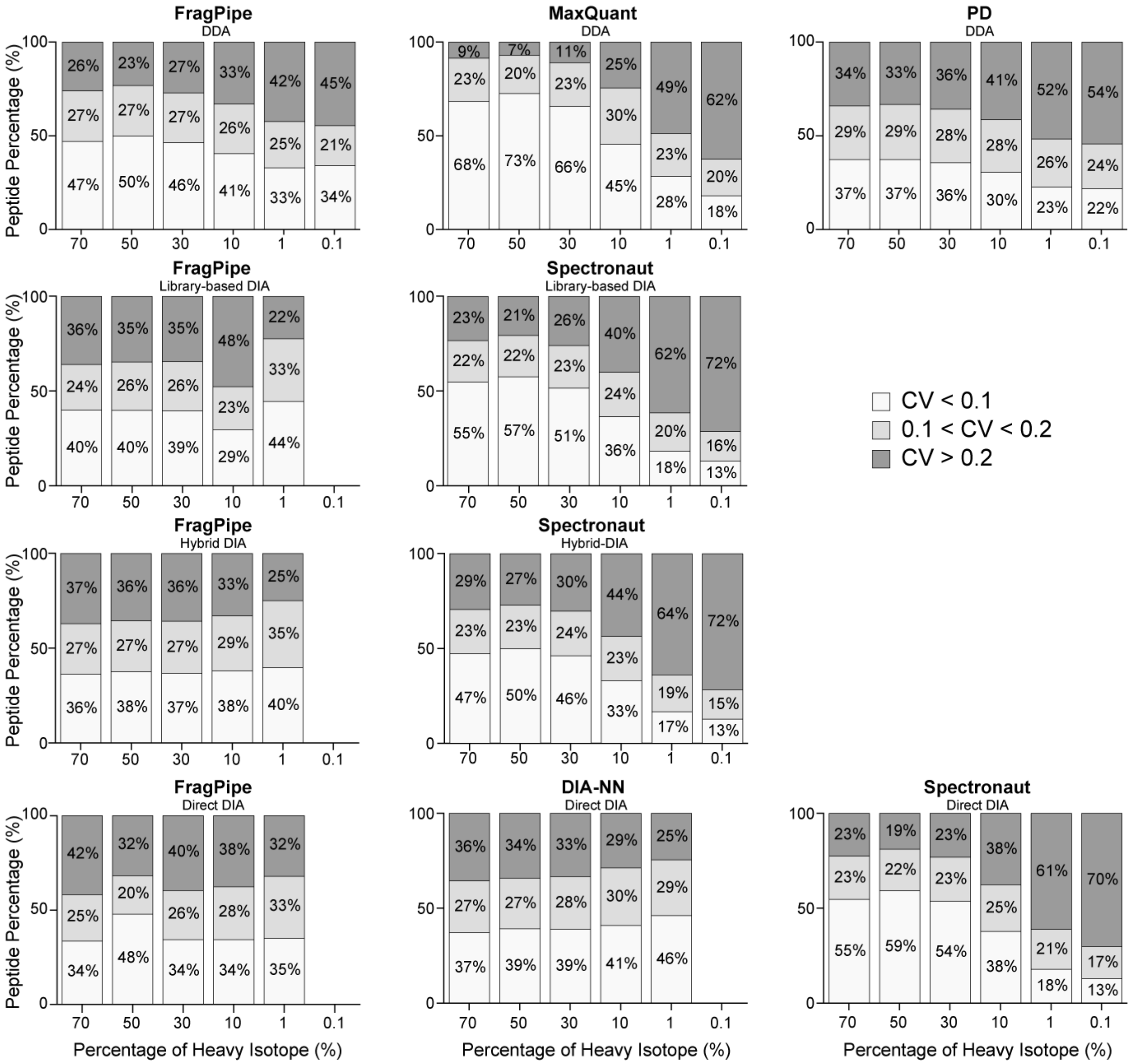

Supplemental Fig. S3: Comparing SILAC quantification precision across technical replicates in different software platforms. Stacked bar graphs showing the coefficient of variation (CV) of three technical replicates with a series of known percentages (70%, 50%, 30%, 10%, 1%, 0.1%) of heavy peptides.

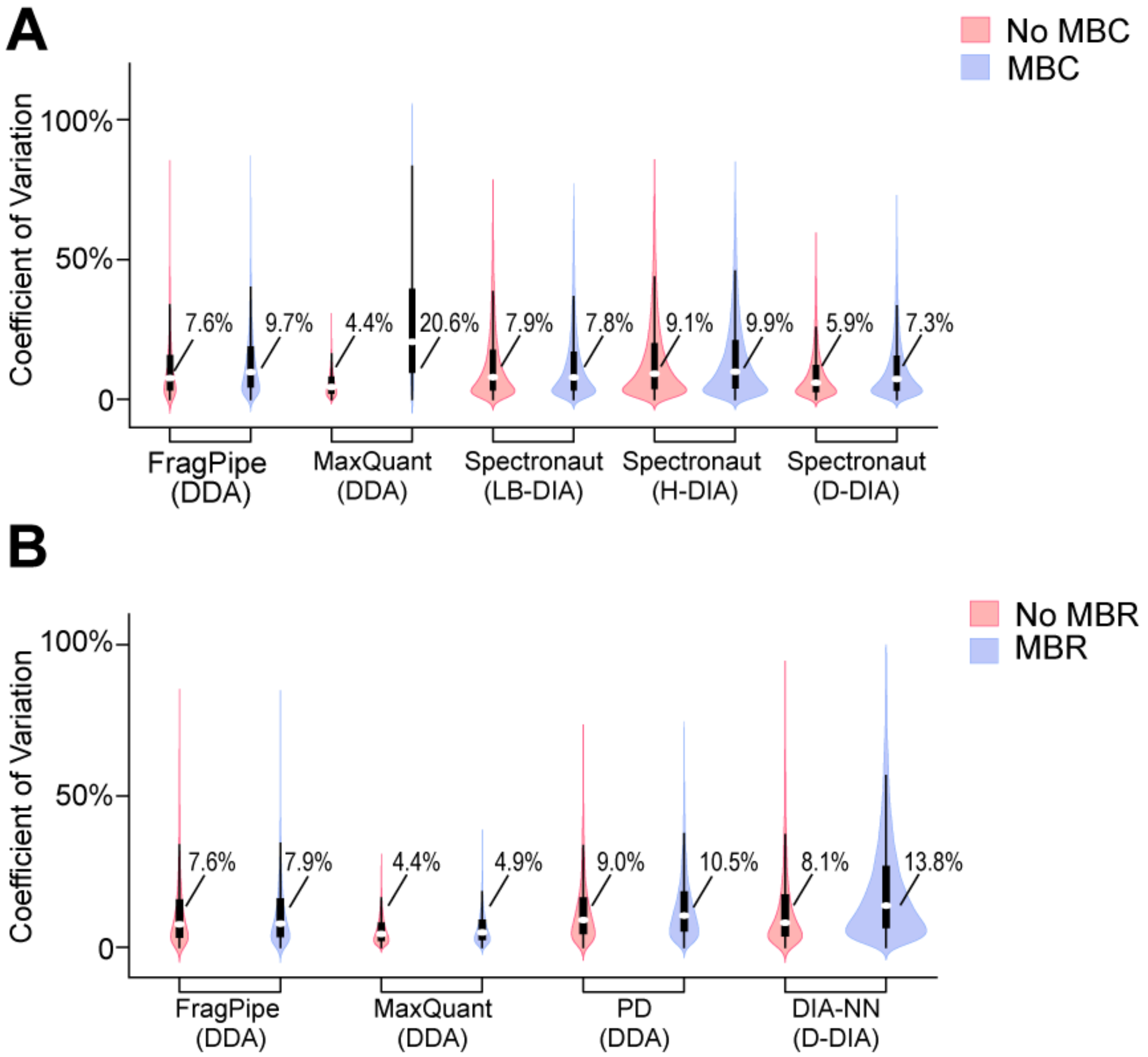

Supplemental Fig. S4: Comparing the quantification variation with and without enabling match between channels (MBC) and match between run (MBR) functions. (A) Coefficient of variation across three technical replicates with and without enabling the MBC. (B) Coefficient of variation across three technical replicates with and without enabling MBR.

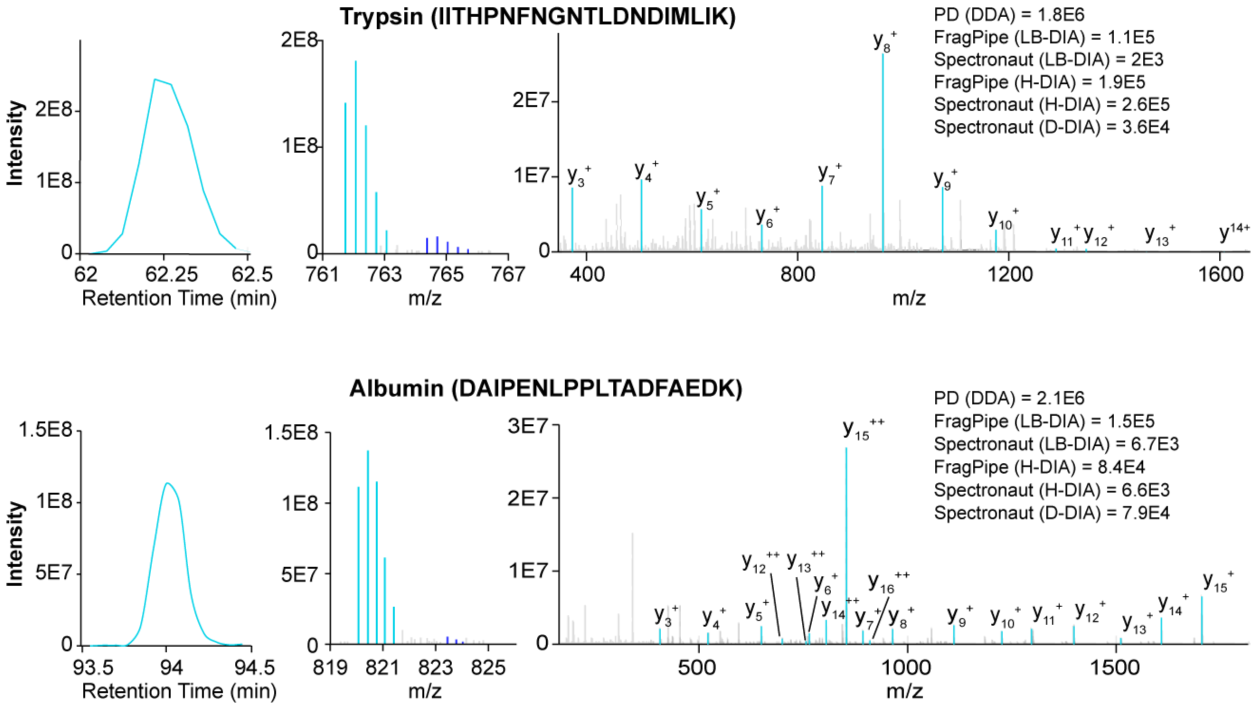

Supplemental Fig. S5: Example false identification of contaminant proteins in SILAC proteomics dataset. LC chromatograms, precursor MS1 spectra and MS/MS fragmentation spectra for contaminant peptides from trypsin (top) and albumin (bottom). Contaminant proteins should only exist in the light version (light blue) in the sample and heavy version (dark blue) represent false identification. The detected heavy peptide intensities (false identification) from various software platforms are denoted on the right.

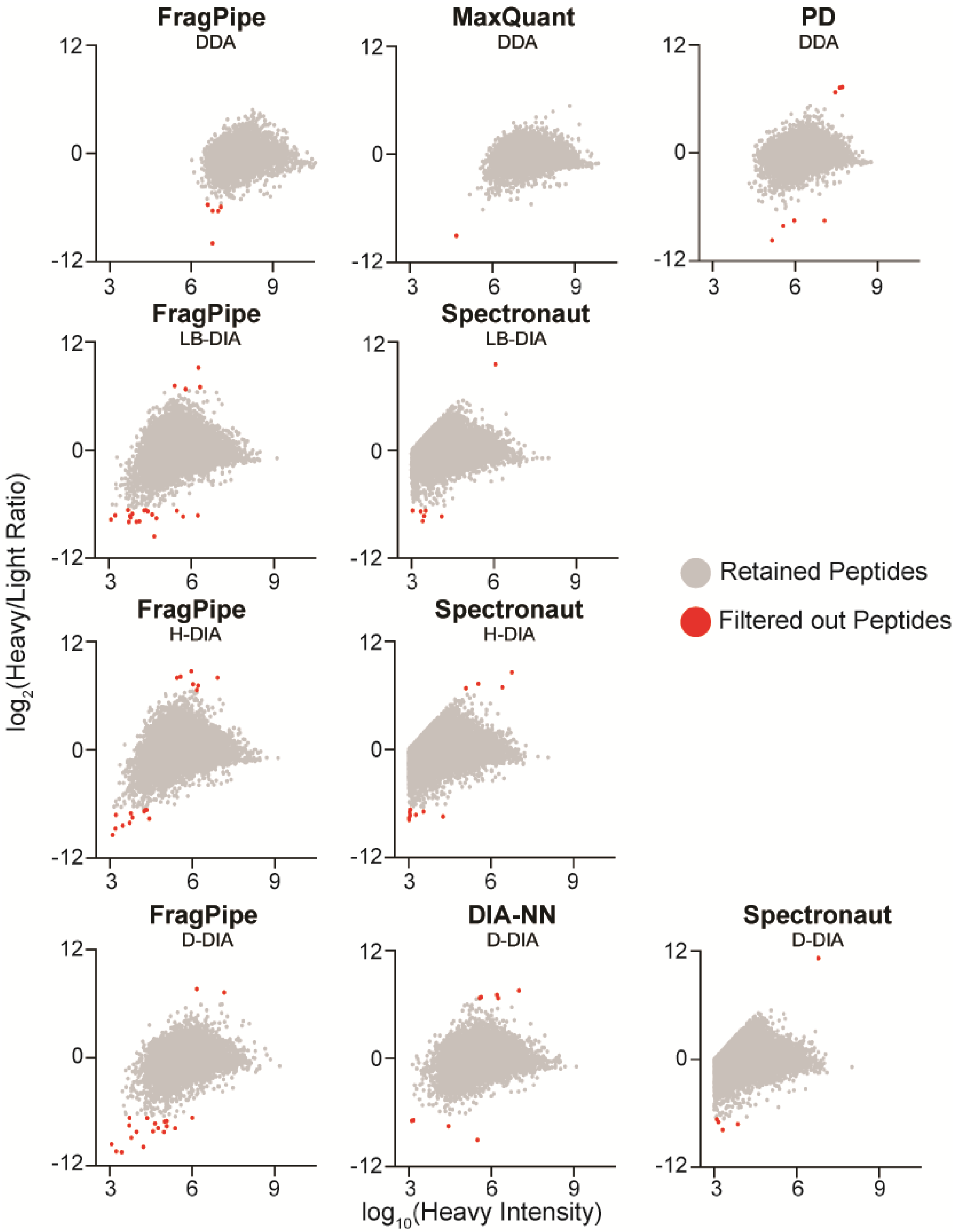

Supplemental Fig. S6: Removing outlier heavy/light ratios from dynamic SILAC proteomics data in different software platforms. Peptides with ratios below 0.01 and above 100 were removed from the dataset before calculating protein half-lives. Removed outlier peptides are highlighted in red.

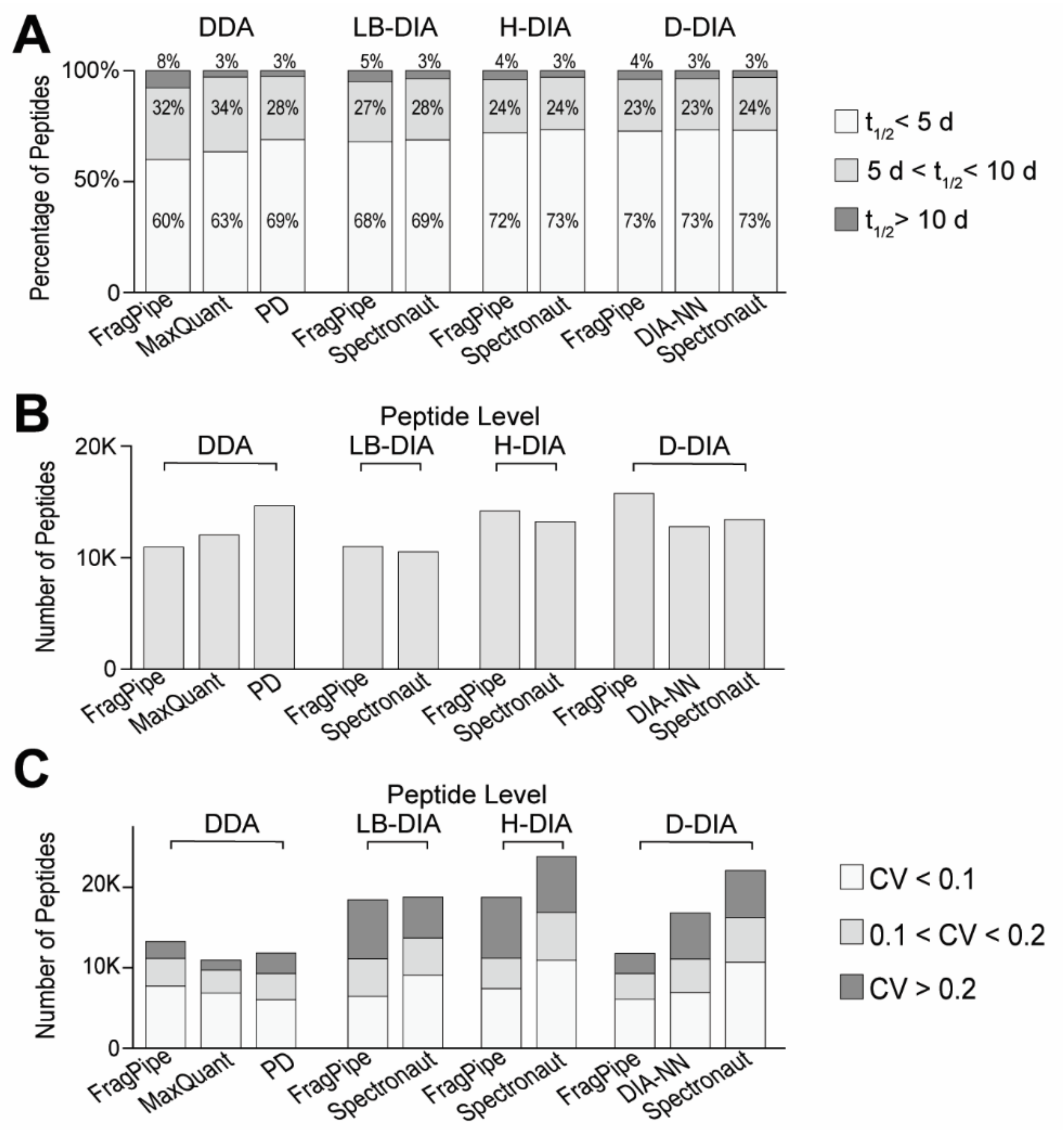

Supplemental Fig. S7: Comparing dynamic SILAC proteomics using different data analysis platforms. (A) Stacked bar graphs showing the distribution of protein half-lives from human iPSC-derived neurons harvested at 1, 2, 4, 6-day time points after switching to the heavy lysine-containing medium. (B) Numbers of peptide half-lives measured from different software. (C) Bar graphs showing the distribution of coefficient of variation (CV) from three biological replicates of dSILAC samples.

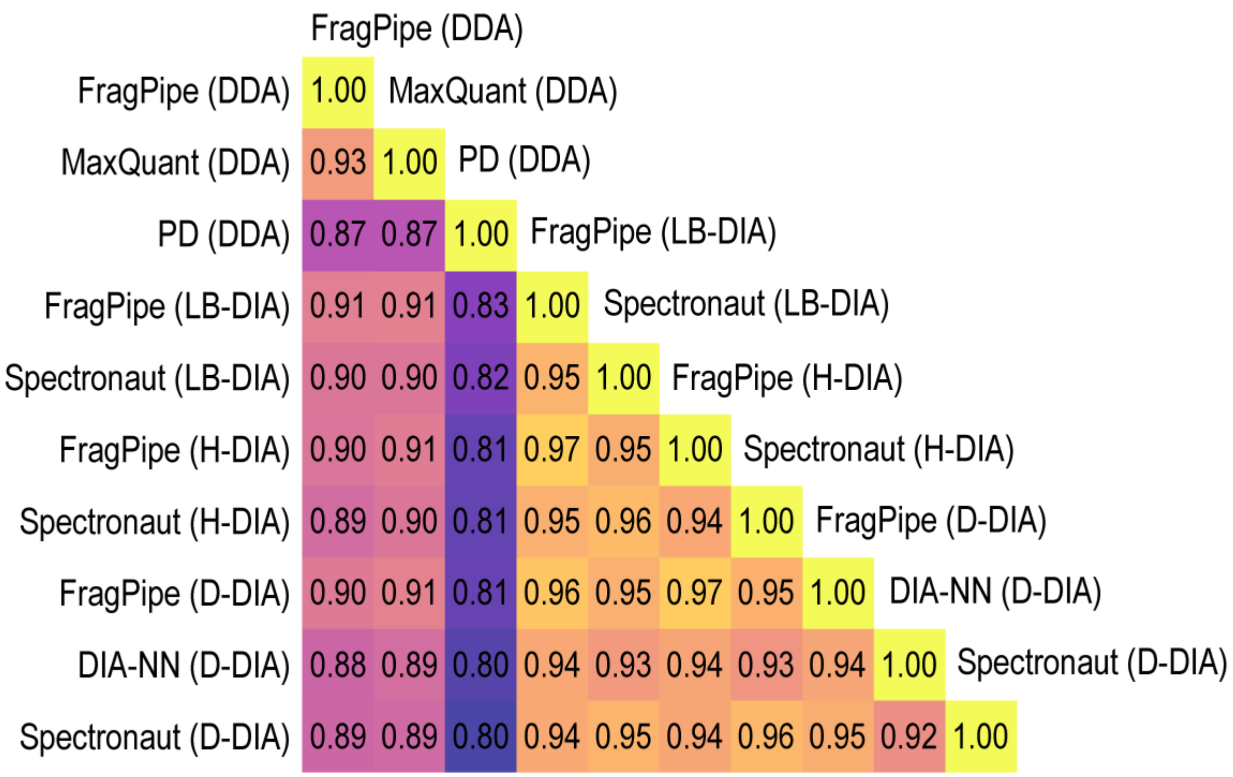

Supplemental Fig. S8: Spearman correlation of global protein half-lives measured using various SILAC proteomics data analysis platforms. Correlation coefficients are labeled on the graph.

Supplemental Table S1: Summary of SILAC proteomics datasets used in this study and generated spectral libraries.

Supplemental Table S1A: Summary of in-house generated and repository SILAC proteomics datasets used in this study. (All datasets have been deposited to ProteomeXchange).

Data Description	Purpose	Source	Cell type	Data type	Heavy labels	Raw files	Proteome Xchange ID
100% light	FDR	Pino et al.	HeLa	DDA, DIA	-	6	PXD022659
100% heavy	FDR	Pino et al.	HeLa	DDA, DIA	Lys8, Arg10	6	PXD022659
Series samples with known heavy/light ratios (70, 50, 30, 10, 1, 0.1% heavy)	Identification & Quantification	Pino et al.	HeLa	DDA, DIA	Lys8, Arg10	36	PXD022659
Dynamic SILAC proteomics with multiple time points (1, 2, 4, 6 days)	Half-life measurement	In-house	Neuron	DIA	Lys8	136	PXD049255
Dynamic SILAC proteomics with multiple time points (1, 2, 4, 6 days)	Half-life measurement	In-house, Hasan et al.	Neuron	DDA	Lys8	16	PXD040251
Dynamic SILAC proteomics from a single time point with three technical replicates	Half-life, Reproducibility	In-house	Neuron	DDA, DIA	Lys8	6	PXD049255

Supplemental Table S1B: Summary of spectral libraries generated in this study.

Data Description	Source	Cell type	Data type	Heavy labels	Raw files	Proteome Xchange ID	Protein IDs	Peptide IDs
Dynamic SILAC proteomics with multiple time points (1,6, 24, 40 h)	Zecha et al.	HeLa	DDA	Lys8, Arg10	96	PXD023218	7,522	98,469
Dynamic SILAC proteomics with multiple time points (1.5 h, 3 h, 6 h, 1, 2, 4, 6 d)	In-house	Neuron	DDA	Lys8	84	This paper, PXD053612	5,172	46,617
Dynamic SILAC proteomics with single timepoint (6 d)	In-house	Neuron	DDA	Lys8, Arg10	23	This paper, PXD053612	6,637	47,992

Supplemental Table S2: Summary of software capabilities, features, and recommended settings for SILAC proteomics data analysis.

Software	Type	Accepted data type	Capability for SILAC proteomics	Recommended settings for static SILAC	Recommended settings for dynamic SILAC
MaxQuant	Free	.raw, mzml	DDA	Enable requantify function	Static SILAC setting; Remove ratios <0.01 and >100, Remove peptides with curve fitting R^2>0.8
Proteome Discoverer	Commercial	.raw	DDA	Not recommended	Not recommended
FragPipe	Free	.raw, .mzml	DDA, LB-DIA, H-DIA, D-DIA	Include requantification; Remove b-ions from quantification for DIA analysis; Remove peptides with abundance <1000, Remove peptides with FDR >1%	Static SILAC setting; Remove ratios <0.01 and >100, Remove peptides with curve fitting R^2>0.8
DIA-NN	Free	.raw, .mzml	DDA, D-DIA	Remove b-ions from quantification; Remove peptides with abundance <1000, Remove peptides with FDR >1%	Static SILAC setting; Remove ratios <0.01 and >100, Remove peptides with curve fitting R^2>0.8
Spectronaut	Commercial	.raw	LB-DIA, H-DIA, D-DIA	Enable "in-silico generation of the missing isotopic channel"; Remove b-ions from quantification; Remove peptides with abundance <1000	Static SILAC setting; Remove ratios <0.01 and >100, Remove peptides with curve fitting R^2>0.8

Note: Table S3 and S4 are provided as separate Excel files.
